# Webcams for Bird Detection and Monitoring: A Demonstration Study

**DOI:** 10.3390/s100403480

**Published:** 2010-04-08

**Authors:** Willem W. Verstraeten, Bart Vermeulen, Jan Stuckens, Stefaan Lhermitte, Dimitry Van der Zande, Marc Van Ranst, Pol Coppin

**Affiliations:** 1 M3-BIORES, Geomatics Engineering, K.U.Leuven, W. de Croylaan 34, BE-3001, Flanders, Belgium; E-Mails: bartvermeulen1@gmail.com (B.V.); jan.stuckens@biw.kuleuven.be (J.S.) dimitry.vanderzande@biw.kuleuven.be (D.V.d.Z.); pol.coppin@biw.kuleuven.be (P.C.); 2 Centro de Estudios Avanzados en Zonas Aridas, Universidad de la Serena Benavente 980, Casilla 599, 172-0170 La Serena, Chile; E-Mail: lhermitte.stefaan@ceaza.cl; 3 Laboratory of Clinical Virology, Rega Institute, K.U.Leuven, Minderbroedersstraat 10, BE-3000 Leuven, Flanders, Belgium; E-Mail: marc.vanranst@rega.kuleuven.be

**Keywords:** bird migration, webcams, object detection, object tracking

## Abstract

Better insights into bird migration can be a tool for assessing the spread of avian borne infections or ecological/climatologic issues reflected in deviating migration patterns. This paper evaluates whether low budget permanent cameras such as webcams can offer a valuable contribution to the reporting of migratory birds. An experimental design was set up to study the detection capability using objects of different size, color and velocity. The results of the experiment revealed the minimum size, maximum velocity and contrast of the objects required for detection by a standard webcam. Furthermore, a modular processing scheme was proposed to track and follow migratory birds in webcam recordings. Techniques such as motion detection by background subtraction, stereo vision and lens distortion were combined to form the foundation of the bird tracking algorithm. Additional research to integrate webcam networks, however, is needed and future research should enforce the potential of the processing scheme by exploring and testing alternatives of each individual module or processing step.

## 1. Introduction

The monitoring of birds has a widespread potential in numerous applications in ecology, climatology, and avian related zoonosis/infections such as avian influenza [1–5]. Migratory birds are known to be carriers of the birds’ flu, caused by type A of the influenza virus H5N1 [2] and they can infect domesticated birds [6,7]. This virus can cause severe disease in humans, but at present it cannot transmit easily from person to person [8], although fatal human cases were reported [7,9]. By monitoring wild bird migration a better understanding of the flyways used by the various avian species can be gained [10–12]. A network of cameras/webcams for reporting of migratory birds can be explored in this context, since webcams and more sophisticated cameras were previously used for other monitoring objectives, such as for instance traffic monitoring, security applications and the military [13].

Various techniques for bird monitoring already exist and some of them are more time-consuming and expensive than others. Direct observation is the simplest and oldest technique and may differentiate migratory birds based on size, color, song and flight characteristics [14]. Slight modifications allow also night observations (moon-watching technique [14,15]) by applying terrestrial vertical light beam or ceilometer techniques [16]. The use of passive infrared cameras (measuring avian body heat) allows cloudy night observation and reduces disturbances due to artificial light [17,18]. Capture-recapture techniques also can provide valuable information about bird migration [14,19] but less than 1% of the non-hunted species are ever recorded again [14]. Radio tracking or telemetry is another method to monitor birds [20,21]. A small radio transmitter is attached to the bird and sends a periodic beep signal which is tracked down. Birds, however, with an attached device have a significantly lower survival rate. More recently this technique evolved to GPS-tracking which rules out some of the limitations of radio tracking [12,22]. Another approach for bird detection is the use of acoustic based technology to identify bird specific signatures such as drums from woodpeckers [23]. Also radar based monitoring technology is used for bird detection, although the coarse spatial resolution is a limiting factor [24].

Novel tools for bird monitoring are using distance retrieval from stereo vision and motion detection. Stereo vision is used in several domains where depth is essential, for instance in robot-computer vision [25], traffic flow control [26] or in ecological studies such as fish size measuring [27,28] or to rediscover extinct birds such as the Ivory-billed Woodpecker (*Campephilus principalis*) in the US [23]. Most methods are based on the knowledge of the disparity and the angle between the cameras and the object [28]. Background subtraction is a common approach to detect moving objects in a sequence of images or videos. It consists of comparing each frame with background model and retaining the foreground pixels that differ significantly from the background. This technique is often implemented in computer vision applications, video surveillances, traffic monitoring and human tracking [30–32].

In this study the potential of permanent cameras such as webcams for bird detection is analyzed. Webcams are low-cost cameras, can be adapted to be robust to weather conditions, and have the ability of remotely-control and unsupervised operation capabilities. As such, they can be used as first analysis tool for tracking moving objects. With this demonstration study, we aim to sensitize readers on the potentials of webcams for a variety of applications of monitoring moving objects. In this context, a measuring protocol is proposed consisting of lens correction, background subtraction, object tracking, and distance or height calculation. This protocol subsequently is used in a basic case study to analyze the limits of this instrument for bird monitoring applications.

The overall aim is to assess the quality of bird or “object” detection by a webcam. More specifically:

- The first objective deals with webcam detection capability, where the detection limits for object velocity, contrast and size are analyzed in relation to the visibility of a bird on a webcam video. This is studied by means of an indoor experimental set-up recording artificial objects, *i.e.*, pearls, attached to a pendulum to mimic flying objects.
- The second objective addresses the webcam tracking capability, where sources of error and their ranges are discussed. Therefore, a simple 3D-model was built linked to processing tools that allocate the correct coordinates to the correct objects.

In summary, an experiment was set up to analyze and process webcam recordings for retrieving information about the flight altitude, direction and velocity of migratory birds.

In the section on Experimental Design, the materials and methods necessary to study the webcam detection capability and the tracking capability are presented. In the section on Results and Discussion the effects of inaccurate position of the cameras, incorrect determination of pixel coordinates of objects and lens distortion are also examined. In the section on Application of the Webcams, we demonstrate the use of webcams in an outdoor experiment. Finally, in the last section, conclusions and recommendations are formulated.

## 2. Experimental Design

### 2.1. Materials

The measuring set up consists of a pendulum experiment, combinations of webcams positioned in a stereo pair and connected to software.

*Webcams*: Logitech Quickcam Express and a Creative Live! Cam Vista IM connected to an enhanced Acer Travelmate 4602 were used for the experimental design. They have a standard resolution of 352 × 288 without interpolation and 640 × 480 with interpolation. The horizontal field of view (HFOV) is approximately 40° and 50°, respectively.

**Figure 1.**
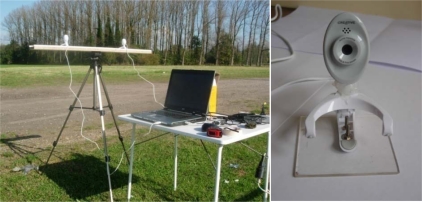
Illustration of the positioning of the webcams for stereo recordings. Left, two Creative webcams. Right, a detail on a Creative webcam mounted on a plate.

*Stereo image recording* requires two webcams, preferentially placed on the same baseline and height, and looking in the same direction. Alternative set-ups would unnecessarily complicate the calculation procedure. The camera’s viewing direction was oriented north to avoid direct sunlight impact on the camera which may result in an over-exposure of the video. Video recordings cannot start perfectly at the same time, so a common marking point must be integrated to ensure synchronous video recordings. This was done, using a sparkling light or a clear visible and distinguishable action in the field of view of both webcams [27]. Figure 1 shows the stereo recording set up as used outdoors.

*Software*. Matlab 7.5.0 [33] was used to acquire videos in the experimental design and to process and analyze the video material. Stereo video acquisitions were done by PY Software’s Active Webcam version 10.1, a surveillance program to perform simultaneous recording and broadcasting from unlimited number of cameras.

**Figure 2.**
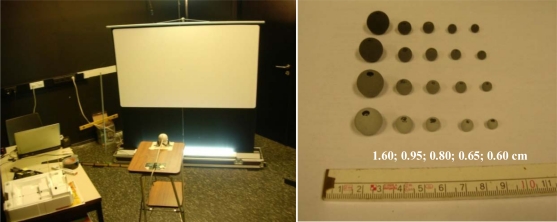
Left: Recording set-up of the pendulum in front of the projection screen and the Logitech webcam. Right: Various sizes (diameter in cm given in white) and colors of pearls used to analyze the effect of size, speed and contrast on the detection capabilities of a simple webcam.

*A pendulum experiment* was conducted to record the visual detection of velocities, contrasts and sizes of objects observed by the webcam. The experiment consists of the Logitech webcam, a Projecta CinemaLite 16:9 projection screen and a pendulum. The pendulum was built up of white Nm 30/3 yarn with a fixed upper end and a leaden weight of 15 g at the bottom end for initiating the movement. About half way the yarn, different sizes and colors of wooden pearls were fixed, one pearl per experiment. Six different diameters of pearls used are indicated in Figure 2.

The initial color of the pearls is black and to obtain three levels of gray they were painted with mixed poster paint. The distance between the pearl and the camera was fixed to 1.40 m to ensure that the full screen fits in the image. The screen was placed 0.20 m behind the pendulum and perpendicular to the camera’s viewing direction. The length of the pearl pendulum was 0.94 m and the lead was fixed at 1.98 m. A vertical bar, that can be moved horizontal and parallel to the projection screen, determined the starting point and thereby the velocity of the pendulum. The projection screen has a white projection surface, whereas the pearls are black to grayish white. To avoid shadows on the projection screen 1,320 Watts of surrounding lights were used. The projectable area of the screen is 88 cm by 146 cm. Recordings were made with a resolution of 352 by 288 pixels at 30 frames per second (fps). Figure 2 illustrates the set-up.

The velocity of the objects was determined as the velocity at the lowest point of pendulum by applying the law of conservation of energy. In the ideal case, the potential energy will be fully converted into kinetic energy (no friction). Five different sizes of pearls were used with four different colors, ranging from black to light grey, and released at six different deviation points (corresponding velocities V1 to V6 are 4.91, 6.02, 6.95, 7.77, 8.51, 9.19 km h^−1^, respectively), resulting in 120 possibilities. These amounts were manageable in the given time frame. Each configuration was acquired five times to rule out possible mistakes. For each object or pearl at 1.40 m distance of the camera a corresponding size on the image can be computed and the real velocity of the pendulum can be converted to image pixel speed. The recordings were analyzed visually to check whether an object could be detected by the webcam in the lowest point on the first sway to know at which velocity, contrast and sizes objects are still traceable.

### 2.2. Measuring and Processing Protocols

**Figure 3.**
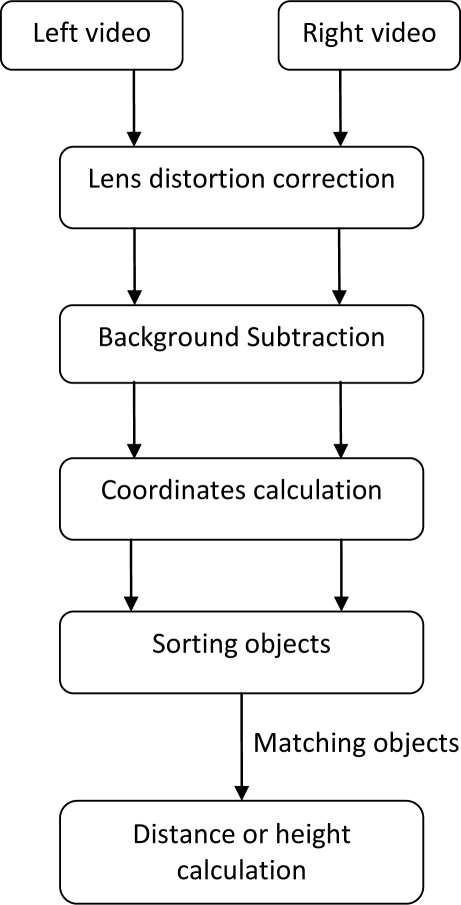
Involved process to calculate distance & height of moving objects.

Prior to analyzing the tracking capabilities of a webcam, the recording must be processed. A schematic overview of the processing steps is given below (Figure 3).

**Figure 4.**
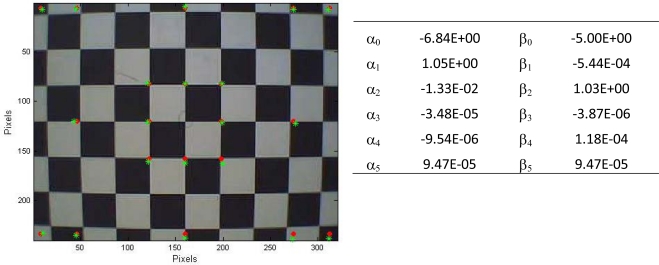
Warping results with the values of the warping equation’s parameters. Red dots are the correct position and green stars are the calculated.

*Lens distortion*. Regular webcams are made for close range recording and for monitoring objects or persons mainly in the centre of the image. Webcams are fitted with a plastic low cost lens suffering from an inherent inaccurate representation of the reality, the so called lens distortion [34]. Hence, prior to deriving metrics from recordings, a lens distortion correction must be performed. Warping equations, transformation of pixels in the input space according to a polynomial equation that is fitted to an amount of control points, can be used to correct the retrieved webcam image. This mathematical relationship is determined by the location of pixels in the image and their corresponding real known coordinates [e.g., 35]. Although more advanced warping techniques are available, a second degree warping was applied in this exploratory study for the sake of simplicity. Correcting lens distortions of a webcam requires a reference panel with known and fixed geometry. A checkerboard is the tool of excellence for correcting these distortions [36,37]. This board was placed in front of the webcam at a known distance perpendicular to the sensor plain. The coordinates of the grid corners, where black and white squares converge, were measured. The pixel size of one square was known thus the actual coordinates of grid corners could be calculated. Both coordinates were put together in a system and solved using singular value decomposition in Matlab. Six is the minimal number of control points required for a second-order warp. Generally, at least the double amount of control point is used. In this research 20 points are used. Figure 4 illustrates the lens distortion, the warping correction and lists the calculated parameters for the polynomial equation. The root mean square error (RMSE) is 1.33 pixels for the x-coordinates and 2.58 pixels for the y-coordinates.

*Background subtraction*. Once stereo videos are made, the coordinates of objects need to be extracted from each frame wherein motion occurs. Therefore, the dynamic foreground and the static backgrounds need to be separated. The simplest form of background subtraction is called the frame difference method which subtracts the current frame from the previous one [38]. A pixel is considered as foreground when the difference in pixels values for that given pixel is larger than a certain empirically determined threshold.

**Figure 5.**
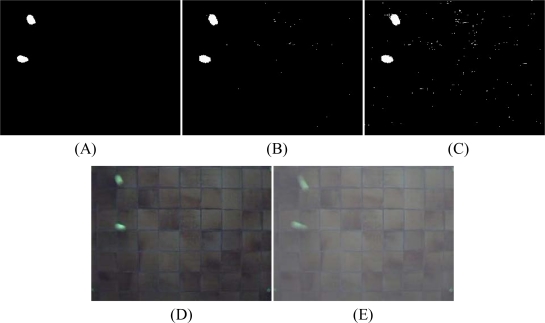
Five images taken with the Creative webcam (resolution 320 × 240) of moving balls on a floor. Images (A), (B) and (C) illustrate a background subtraction with threshold values of 0.050, 0.020 and 0.015, respectively which causes noise. The large white objects are the moving target objects. Noisy pixels are not retained by the threshold and appear as white flecks. Without the noise removal these pixels are falsely classified as moving objects. (D) Deformed object. (E) Overlay of 2 succeeding images.

An issue with motion detection is that objects need to be continuously moving. In frames where objects stay still for more than a frame period (1/fps), no motion will be detected and the corresponding pixels will become part of the background [31,38]. Another background subtraction method makes use of a median filter. The background is determined by the median of the previous N frames. In turn, the foreground of the current frame is the difference between the current frame and the background. Hence, both fast and slow moving objects can be detected just as long as they move a few pixels in a given time frame. This is an advantage compared with the frame difference method. The median filter was used as a jumping window *i.e.*, it was held constant for a number of succeeding frames to reduce memory usage [31]. A median filter is less influenced by outliers than a mean filter. Since this is an exploring study, more advanced filters have not been tested. The background detection algorithm applied in this study is a median filter to search for moving foreground objects. Objects are extracted from the background by an arbitrarily determined threshold. Figure 5 shows three different threshold values: 0.050, 0.020 and 0.015 (gray values scaled between 0 and 1). Since the frames of the recordings are converted to grayscale by summing the RGB-values, the threshold values needs to be multiplied by 256*3. With an inaccurate threshold value, more noise removal has to be applied. As shown in Figure 5, the ‘detected’ object has a slightly different shape than the real object. Two major reasons for this anomaly can be identified. First, fast moving objects are often captured as if there are two objects or one elongated object due to the deformation as illustrated in Figure 5D. Each of the two balls is deformed in such a way suggesting that each ball is composed of two objects or one elongated object. Thus, a fast moving ball is captured on the webcam as one large elongated ball which clearly is an artifact.

**Figure 6.**
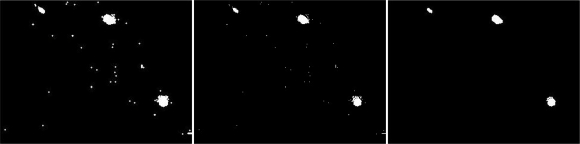
Left, the application of the Matlab dilution algorithm; Middle, normal; Right the application of the erosion algorithm.

A second reason is the appearance of “ghosts” [30,31]. The median filter remembers the old location of the object and produces a ‘ghost’ object behind the target object. Fast-adapting median filters, which has lower buffer sampling rate, are less susceptible for this problem than slow-adapting filter. Background subtraction results in a binary image, *i.e.*, changed pixels and non-changed pixels. In order to remove noise and to stress and merge objects which are placed close to each other erosion and dilution Matlab functions were used (Figure 6). Noisy pixels are not restrained by the threshold and appear as flecks [33].

*Coordinates are determined* by the clumping indexing algorithm in Matlab [33]. This function labels connected components in binary images and returns them in a matrix together with the number of components. The elements of the returned matrix are integer values larger than or equal to zero. The pixels labeled 0 are the background. The pixels labeled 1 make up one object. The pixels labeled 2 make up a second object, and so on. Out of the matrix, the position of the center of the objects can be derived and used for further processing.

*Sorting objects.* The temporal analysis of an object requires that its changing coordinates can be estimated. When multiple objects in a frame occur, however, it is necessary to assign the right coordinates to the right objects. An image is represented as a matrix where the row number indicates the frame number and the columns contain the coordinates for *n* detected objects. Assume that in a certain frame (time t_i_) two objects are visible with these coordinates:

(1) $$\begin{array}{l} O_{1,(\mathrm{ti})}:(x_{1};y_{1})\\ \;O_{2,(\mathrm{ti})}:(x_{2};y_{2}) \end{array}$$

The predicted position of this object in the following frame (time t_i+1_), assuming linear motion, is:

(2) $$\begin{array}{l} O_{1p,(ti+1)}=O_{1,(\mathrm{ti})}+O_{1v,(\mathrm{ti})}\\ O_{2p,(i+1)}=O_{2,(\mathrm{ti})}+O_{2v,(\mathrm{ti})} \end{array}$$

with *p* for projected position and *v* for speed and *t* for time.

In the next frame the predicted position of the *n* objects is compared to positions of *m* (objects need to be detected since it is not necessarily the same amount) in that frame in a *n* × *m* matrix. Assuming that there are three objects, the object the closest to O_1p_ will be selected using Euclidean distances. If this distance is smaller than a certain threshold distance, the related object is the continuation of the first object. If not, a new object is entering the pathway list. The same is done for the remaining objects of the current frame. Next new projected positions are calculated with knowing speed of the objects e.g.:

(3) $$O_{1v,(\mathrm{ti}+1)}=O_{1,(\mathrm{ti}+1)}-O_{1,(\mathrm{ti})}$$

This strategy continues until all coordinates in each frame are assigned to a specific object. The threshold distance is a combination of a constant and a fraction of the speed of the object e.g., 3 + ½ O_1v_ and stipulated by trial and error.

**Figure 7.**
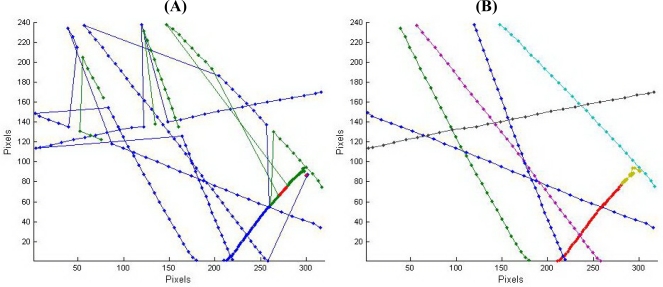
The effect of the sorting algorithm, (A) unsorted object tracings and (B) sorted object tracing. Objects in (B) are aligned and separated from each other, whereas in (A) connections between objects exist.

Coordinates of objects were determined by a row-by-row search of components using the label algorithm of Matlab [39]. Therefore, a sorting process was needed to assign the right coordinates to the right objects. Figure 7A shows the effect of the sorting algorithm of unsorted object lists in a graph, whereas in Figure 7B, the objects are aligned and separated from each other (no connection lines between the object exist). Threshold value determination depends on the frame rate of the recording and the speed of the objects. Objects that are not detected anymore for one or more frames are deleted and will be considered as a new object by the sorting algorithm when it appears again. To deal with this issue, the last known object coordinates is copied for a few amount of frames. Consequently, the object shows up again and the original pathway continues. As such, it is possible to calculate distances which are crucial in the sorting algorithm.

**Figure 8.**
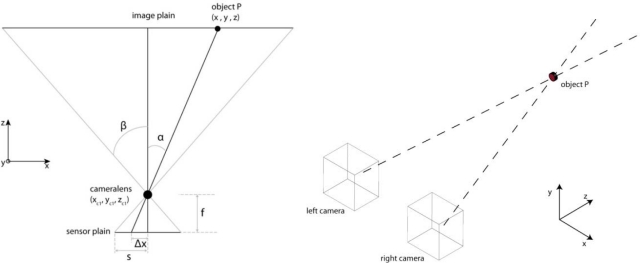
Left: Model and detail of camera (Left); Right: stereo vision.

*Distance calculation*. The calculation of the distance or height of moving objects requires a modeling approach. Our method assumes a pinhole camera to apply triangulate geometry [34]. With the image plain and sensor plain parallel the following equations applies (Figure 8):

(4) $$\frac{s}{\Delta x}=\frac{\text{tan}\;\beta }{\text{tan}\;\alpha }=\frac{s_{\mathrm{px}}\;P_{\mathrm{xdim}}}{\Delta x_{\mathrm{px}}\;P_{\mathrm{xdim}}}$$

(5) $$a=\text{tan}\;\alpha =\frac{\Delta x_{\mathrm{px}}}{s_{\mathrm{px}}}\times \text{tan}\;\beta$$

where β is half the field of view and α the angle between the object and the perpendicular line on the image plain starting from de camera lens. Δx is the difference between the middle and the projection in x-direction. These equations can be applied in both the horizontal and vertical plane.

In 3D, an object is located on the intersection of two lines in space, one for each camera of the stereo vision pair (Figure 8). Equations (6) to (9) show the definition of such a line counting for one camera. This is resulting in a system of four equations and three unknown variables x, y and z:

(6) $$x-x_{c1}=a_{1}(z-z_{c1})$$

(7) $$y-y_{c1}=b_{1}(z-z_{c1})$$

(8) $$x-x_{c2}=a_{2}(z-z_{c2})$$

(9) $$y-y_{c2}=b_{2}(z-z_{c2})$$

where x_c1_, y_c1_, z_c1_ and x_c2_, y_c2_, z_c2_ are known coordinates of camera 1 and 2, respectively, and a_1_, a_2_, b_1_, b_2_ are respectively the slopes in the two perpendicular plains.

This system of equations can be solved using singular value decomposition. Before implementing the coordinates in the system, the lens distortion correction was applied. The z-coordinates give the distance to the object in a plane along the viewing direction. If the height is required or the webcams are placed under a certain angle, corrections for this approach have to be made.

### 2.3. Sources of Errors

Different sources of errors due to model structure, algorithm boundary conditions and observation qualities exist. In this study, the effect of the inaccurate positions of the cameras (camera shift and rotation due to the fragile camera heads), incorrect determination of pixel coordinates of objects and lens distortions were examined. In the stereo vision, multiple possible sources of errors are observed: position shifts, webcam rotations and the incorrect determination of pixel coordinates of objects e.g., not the real center of an object in determined.

*Camera shift and rotation*. The effect of inaccurate positioning of the cameras was tested by means of a position shift of 1 and 5 cm along the baseline and a rotation of one webcam from 0 to 5 degrees for various baseline lengths. The statistical analyses were performed in R (version 2.8.1). The Mann-Whitney U non-parametric test was used to test the difference in error of distance for a baseline length of 1 and 2 m. The effect of inaccurate placing of one webcam decreases when the distance between the cameras increases (p < 0.01). The further the cameras are placed from each other, the more precise the distance calculation is.

*Pixel determination*. Another possible error originates from an incorrect determination of pixel coordinates of objects, e.g., when the real center of an object is not determined. Therefore, pixel deviations of 0.5 and 1 pixel in x and y direction are assumed to quantify the effect of incorrect pixel locations. This will create a raster of 9 points, leading to 81 combinations to investigate. Four distances between the cameras were used to assess the deviations: 2, 4, 6 and 20 m. The simulated object was located at 40 m.

## 3. Results and Discussion

### 3.1. The Webcam Detection Capability

**Figure 9.**
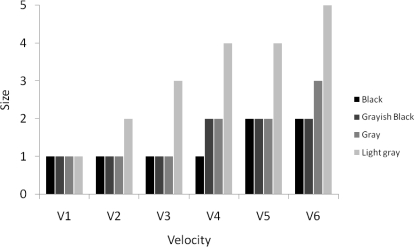
The minimal detectable object size (0.60, 0.65, 0.80, 0.95, 1.60 cm) with a webcam recording at a given object speed (increasing from V1 to V6, or 4.91, 6.02, 6.95, 7.77, 8.51, 9.19 km h^−1^, respectively) and color (black, grayish black, gray, light gray).

Results of the pendulum experiments are summarized in Figure 9, which shows at what size an object is still detectable. Larger objects remain visible at larger velocities than smaller objects. Darker objects are visible longer than lighter objects. Table 1 shows the maximal contrast or intensity difference, an average of five repetitions, in the frame where the pendulum is at its lowest point (*i.e.*, at maximum velocity). Contrast values decrease when the color becomes paler (from black to white). In addition, size 5 (1.60 cm) of the balls shows larger contrast compared to the other sizes.

The importance of the effect of velocity, size and contrast on the detection capability of objects in video recording is reported by several studies dealing with object tracking and motion detection. Intensity or contrast is a common used threshold determinant in background modeling [31,32]. Reference [40] developed a vehicle tracking algorithm based on the combination of a per pixel background model (an extension of work by [41]) and a set of single hypothesis foreground models based on object size, position, velocity, and color distribution. Instead of using grayscale video, [31] argued that color image, either red-green-blue (RGB) or hue-saturation-value (HSV) color space, is becoming more popular in the background subtraction models. In addition, [30] and [42] state that color is better than intensity or luminance at identifying objects in low-contrast areas.

### 3.2. The Webcam Position

**Figure 10.**
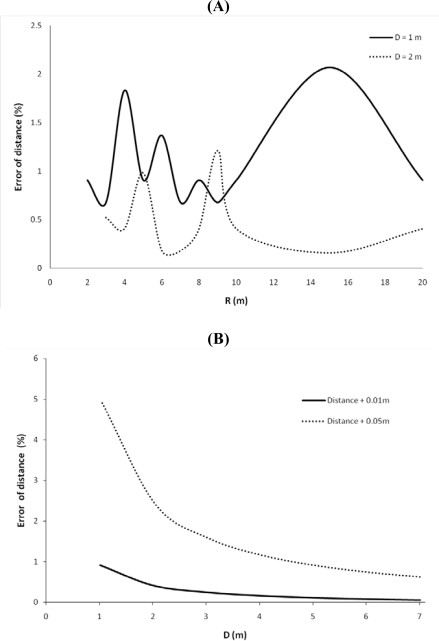

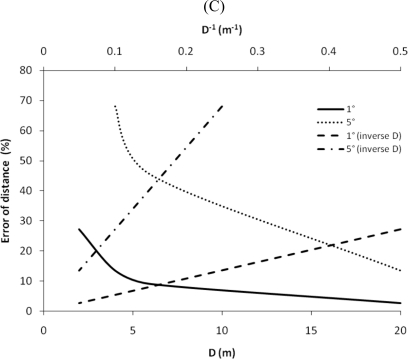
(A) Relationship between the distance and the calculation error on the distance due to a position shift (1 cm) of one camera for baseline length D of 1 and 2 m. (B) Relationship between baseline length D and the calculation error of the distance due to a position shift of 1 and 5 cm of one camera for an object placed on 20 m. (C) Relationship between baseline length D and the calculation error of the distance of the object located on 40 m with a rotation error of 1 and 5 degrees. A linear relation is observed with the inverse distance D.

Figure 10 illustrates the relationship between the distance and the calculation error due to a position shift. The fluctuations of the curves in Figure 10 are likely due to the discretization process. Similar results were found by [43–45]. Reference [43] developed a ‘multiple baseline stereo method’ which combines the advantages of a large and a small baseline length. The advantages are a high depth accuracy and faster image overlapping procedure, respectively. For similar reasons, [44] developed a multi viewpoint linking’ method. A variable baseline stereo tracking vision system was designed by [45]. The system uses a high-speed linear slider to adapt the distance between cameras to improve the accuracy of 3D estimation, especially when dealing with fast moving objects.

The effect of a horizontally rotated webcam, which results in the wrong determination of coordinates, decreases when the distance between the webcams or baseline length increases (Figure 10C). A horizontal rotation of 1 degree results in an error of the distance of about 27% for inter-camera distance of 2 m, whereas a 5 degree rotation results in an error of 68% with a baseline length of 4 m. Comparison of the 2 m baseline length for both rotations was not possible since there was no overlap with the 5 degree rotation. The object was placed on 40 m in this error assessment. In conclusion, the larger the distance between the cameras, the smaller the distance error, but also the smaller the stereo overlap.

**Figure 11.**
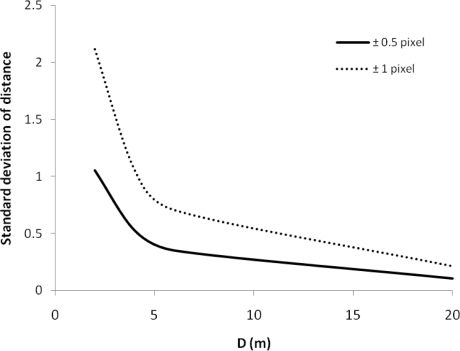
Relationship between baseline length D and the standard deviation of the calculated distance for pixel deviations of 0.5 and 1 pixel. The standard deviation decreases with increasing distance D and is larger for a larger pixel deviation.

A close to normal distribution is observed in the histograms of the standard deviation of the calculated object distance (Figure 11). The standard deviation decreases when the baseline length increases (Figure 11). Similar results were found by [43,44].

Since lens distortion is present in all the webcams’ image recordings, a correction was necessary. A checkerboard was positioned perpendicular to the sensor plain at known distance. The control points used to determine the warping equations are given in Section 2.2.

If no correction for lens distortion is applied, objects, especially those at the edge of the image, are placed inwards on the image creating incorrect pixels and inaccurate distance calculation (see also section 3.2.6.). A study of plumb-line calibration in reference [46] showed an RMSE between 1 and 1.7 pixels depending on the quantity of used lines. The larger the number of straight lines measured, the smaller RMSE values were found. Reference [47] obtained an average error of less than 0.3 pixels in an image with a resolution of 1,024 × 768 pixels with a calibration algorithm based on Taylor series expansion. Several more advanced digital calibration methods were compared by [48].

### 3.3. The Webcam Tracking Capability: a 3D Model for Tracking Moving Objects

**Figure 12.**
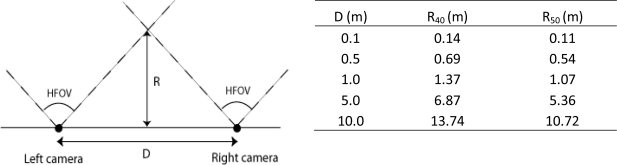
Left: Illustration of stereo vision and overlapping point with D the baseline length, R the distance to the overlapping point of both camera views having an angle HFOV. Right: Distance R to the point where overlap occurs with stereo vision for cameras with HFOV of 40 (R_40_) and 50 degrees (R_50_) for specific D values.

Two ideal pinhole cameras (Figure 12), looking at an object with known coordinates, were simulated using the POV-ray program (Persistence Of Vision ray tracer program) [49]. With this program, images are captured as if the camera is looking to real objects. The simulations demonstrate a maximal model error in z-direction (height) of 1.37%, an average of 0.26% and a standard deviation of 0.27%. The error increases when the object is placed in the middle between the cameras.

Higher error values in z-direction, are likely due to the discretization of the data in raster images, *i.e.*, the conversion of continuous variables to discrete ones. For instance, when the object is positioned on a location with real coordinates, it will be converted into a discrete value (for example (2.3949, 8.8434) into (2,9)). A Monte-Carlo analysis [50,51] probably will improve the model’s uncertainty assessment in future work. The error on z coordinate does not change considerably with increasing distance of D. Since images of both cameras do not overlap at close distances, simulations were started at particular object distances shown in as Figure 12. The point where the two fields of view converge depends on the inter-camera distance and the HFOV.

## 4. Application of the Webcams

Since the proposed processing algorithm (see Figure 3) was developed with emphasis for bird migration monitoring application as introduced in the first section, some considerations on bird flight characteristics are required. The knowledge of features such as flight altitude and velocity, and the size of individual species is important, especially on Anatidae (ducks, geese and swans) and Charadriiformes (waders and gulls) which are the natural host of the avian influenza virus [12]. Table 2 summarizes the length and wingspread of some of the most important hosts of the avian influenza virus.

The applicability of webcam monitoring is demonstrated with an example. Flight altitudes of most common migratory birds over land show a large variation but are typically below 1,500 m [53].

Table 3 gives the number of pixels that represent a meter at various distances R for common resolutions. The values differ a factor 3.2 between the lowest (320×240) and the largest (1,024 × 768) considered webcam resolution. Suppose a Bar-headed goose (L = 75 cm, S = 150 cm from Table 2) flying perpendicular over a webcam at 500 m height, then the bird will occupy approximately 1.035 by 0.293 pixels (0.69 pixels × 1.50 m and 0.39 pixels × 0.75 m) and 3.3 by 0.93 pixels in case of a resolution of 320 × 240 and 1,024 × 768, respectively, according to Table 3 and under the assumption that the bird is flying along shortest axis of the image. The maximum flying height of birds (for instance bird species as mentioned in Table 2), which can be detected by a webcam, depends on many factors. Key variables are bird size and distance and the spatial resolution of the sensor.

Besides the flight characteristics of birds, the color of the plumage is also an important feature since the contrast between plumage and background will considerably determine the detection capacity of the webcam recording. More about the importance of contrasts can be found in [31,32,40] for intensity and RGB color space in [30,42].

Apart from the difference in contrast between birds and sky (or any other background), also the light intensity and the sensor signal-to-noise-ratio play an important role for bird detection purposes. Moreover, also the application that is aimed at is a factor that should not be ignored. Counting birds differs significantly from bird identification or from tracking individuals or groups of birds. Some group of birds may have characteristic flying configurations, which are relatively easy to recognize. In case that the minimum size of a bird can be assumed as the determining factor to detect birds, birds with lengths that correspond with one pixel might be detected (although sub-pixel objects can be detected if the contrast between object and background is high enough). Thus, combining this assumption with the numbers from Table 2 and Table 3 for a webcam with a resolution of 320 × 240 pixels, the maximum height of the birds should not exceed 30, 50, 92, 104, 115, 144, 183, 289 m for respectively the Little ringed plover, Common redshank, Wigeon, Eurasian curlew, Western gull, Bar-headed goose, Grey heron, and Mute swan (Table 2). These numbers are obtained by multiplying the amount of pixels of Table 3 (vertical resolution) with the bird length and then recomputed to one pixel. For the Bar-headed goose example the calculations are: 0.75 m × 193.01 pixels = 144.75 m for R = 1 m. Since the distance and the amount of pixels is inverse proportional, the values for R = 1 are the corresponding values for the height of birds. In case webcams with better resolution are used (for instance with 1,024 × 768 pixels or even higher), a dramatic increase of the maximum height for the detection of the before-mentioned birds can be obtained, respectively 98, 160, 296, 333, 370, 463, 586, and 926 m. These numbers might suggest that webcams can be useful for detection or tracking of bird species flying at these altitudes. Hence, close range birds are the main target group for the use of webcam technology. However, since this technology evolves quickly and to date, already webcams of two megapixels are available, far range bird detection might become feasible in the nearby future.

**Figure 13.**
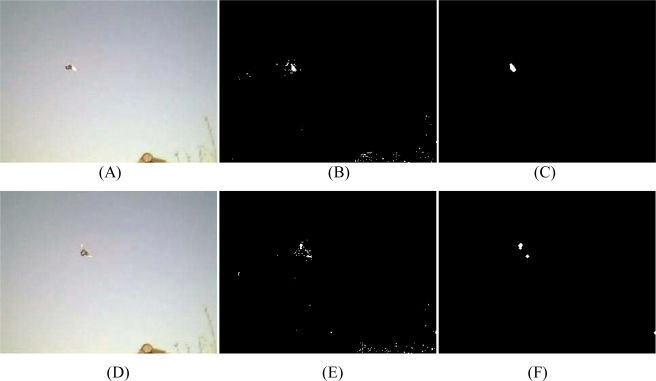
Processing example of two succeeding frames of a webcam recording a flying pigeon. (A) and (D) show the full color image; (B) and (E) the outcome of the median filter, and (C) and (F) after processing to reduce noise and to connect foreground pixels to a number of adjacent foreground pixels. Due to wing flap, two objects emerge.

Analyzing outdoor webcam recordings of a flock of pigeons flying at low altitude nearby a pigeon house revealed some new issues. Due to fast bird flapping, in some frames the wings are detected as two separated objects causing additional difficulties for the proposed sorting algorithm (Figure 13A–F). Decreasing the threshold in the background subtraction phase can reduce this issue, but makes it more sensitive to noise. This anomaly only occurs in case of close-range recordings when more details of a bird are captured (no longer considerable as a point or circle).

**Figure 14.**
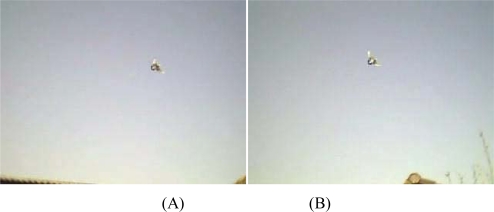

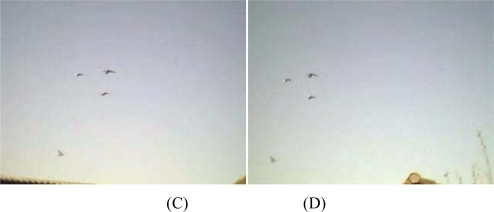
Two stereo pairs of airborne pigeons. Model application revealed that the birds were flying at approximately 6 m ((A) & (B)) and 4.5 m ((C) & (D)) altitude, respectively.

Manual stereo matching delivered following results: the flight altitude of the bird shown in Figure 14 is between 5.5 and 6.7 m calculated for 5 stereo matching pairs. In similar recordings the birds were estimated to fly on 4.5 m height (Figure 14). In both cases the webcams were lined up at 1.205 m height with the baseline length of 1.560 m as illustrated in Figure 1.

## 5. Conclusions and Recommendations

An exploratory and demonstrative study was performed to assess the potential use of webcams for monitoring bird migration posing two research questions: (i) what is the detection capability of moving objects in webcam recordings; (ii) and how can birds be tracked and followed in these recordings?

Webcams are low-cost cameras with the ability for remotely-control and unsupervised operation that in addition can be adapted to be robust to weather conditions. As such, they have potential as first analysis tool for bird migration. With this demonstration study, we aim to stimulate and incite readers on the potential use of webcams for a variety of applications. It is a call for the standard implementation of low cost and unsupervised operational tracking technology. In order to demonstrate this, an experimental design was set up to study the detection capability using objects of different size, color and velocity recorded indoor on a white screen. The results of this experimental set-up indicate the minimum size, maximum velocity and required contrast of the objects. In order to track and follow moving objects with different velocities, a processing scheme was introduced. A background subtraction model separates the moving foreground objects from the static background. To ensure that the right coordinates are matched with the right target objects, a sorting algorithm was developed. Since webcams have low cost plastic lenses with an inherent inaccurate representation of the reality, a correction for lens distortion was performed by a second degree warping. Stereo vision was implemented to create depth vision and to calculate the distance between the object and the cameras in order to determine flight altitude, direction and speed.

Further research should focus on potential alternatives of the current modular processing. Each individual processing step works independent of the modular approach and next step and thus can be replaced by one that has better accuracy or precision. In the future, it is likely that webcams with higher resolutions will become available at lower prices. As such, this would increase details in webcam recordings and optimizing detection capabilities. Literature described various background models and motion detection methods that are worthwhile to be investigated further for their possibilities and potentials. The development of webcams, specially adapted to work in stereo vision, could ease the adjustments and make recordings more precise. In addition, further thinking about the following issues aiming at long term goals is needed:

- Establishing a complete set-up of webcams in the field in order to collect temporal and spatially distributed data. In such detailed studies the practical advantages, disadvantage, limitations and related technical issues of webcams can be revealed;
- Constructing and participating in a network of multiple cameras to cover larger areas (line up, raster); these webcams can be integrated in existing measuring networks of for instance biophysical parameters of vegetation [54,55];
- Communication between cameras and a central data collection station (wireless);
- Real-time processing;
- Preferential hotspots to locate the networks (migratory pathways);
- Participation of volunteers (internet);
- Feedback to participants (internet);

Within an extended network, webcams might be an important instrument against the distribution of avian influenza since it can be used to detect and track birds with a spatial and temporal resolution depending on the network density and webcam design. As such, spots with increasing amount of birds which potentially may be carrier of the avian influenza virus can be located in a dynamic way. With the current easy available webcams, however, the identification of bird species remains an issue. Better knowledge of flyways and staging areas of birds can for instance contribute in assisting policy makers for taking timely and promptly measures in order to prevent or slow down the transfer of avian influenza to domestic birds. In turn, this may help in reducing the risk of virus transfer to humans.

Besides monitoring bird migration, the processing scheme can also be used in other topics such as bird migration changes linked to global warming. With the current easy available webcams, however, the identification of bird species remains an issue. From this demonstration study, considering the quantitative examples of section 4, it can be concluded that the major utility of webcams is not necessarily large-scale migration patterns, but rather to monitor the movement of birds at lower flying altitude in specific areas such as airports, wind farms and at specific staging areas where birds shelter, forage and/or mate. However, since webcam technology evolves quickly and to date, already webcams of two megapixels are available, far range bird detection might also become feasible in the nearby future. Subject to small adaptations, webcams can also be useful in other small scale ecological surveys where the detection and tracking of moving objects is targeted, for instance the capturing of the behavior of wild and domesticated animals (*i.e.*, monitoring and guarding sick or pregnant animals in stables).

## Figures and Tables

**Table 1.** The maximum contrast or intensity difference (gray scales 0–255) in the frame where the pendulum is at maximum speed for each size S (1.60, 0.95, 0.80, 0.65, 0.60 cm), velocity V (4.91, 6.02, 6.95, 7.77, 8.51, 9.19 km h^−1^, respectively) and color (black, grayish black, gray, light gray).

**Size**	**S1**	**S2**	**S3**	**S4**	**S5**	**S1**	**S2**	**S3**	**S4**	**S5**
**Velocity**	*Black*					*Grayish black*				
**V1**	78	78	79	77	110	78	77	77	77	106
**V2**	77	78	78	77	94	78	77	78	77	82
**V3**	77	77	77	78	86	78	77	78	78	80
**V4**	77	78	77	78	81	78	77	78	78	79
**V5**	77	79	78	78	78	78	77	77	79	78
**V6**	79	79	80	79	77	80	80	79	80	78
	*Gray*					*Light gray*				
**V1**	77	77	77	77	91	77	76	77	76	93
**V2**	78	77	77	77	82	78	77	76	77	76
**V3**	77	77	77	78	78	78	77	77	77	76
**V4**	77	78	78	78	77	77	77	77	77	77
**V5**	78	77	76	77	78	77	76	78	77	77
**V6**	79	77	81	79	79	77	76	78	76	77

**Table 2.** Length L and wingspread S [49] of some of the most important hosts of avian influenza according to FAO [12].

**Scientific name**	**English name**	**L (cm)**	**S (cm)**
*Anas penelope*	Wigeon	48	80
*Anser indicus*	Bar-headed goose	75	150
*Cygnus olor*	Mute swan	150	220
*Numenius arquata*	Eurasian curlew	54	-
*Tringa totanus*	Common redshank	26	-
*Charadrius dubius*	Little ringed plover	16	-
*Larus occidentalis*	Western gull	60	-
*Ardea cinerea*	Grey heron	95	185

**Table 3.** Number of pixels that represent a meter at various distances R for common resolutions: 320 × 240, 640 × 480, 800 × 600 and 1,024 × 768 pixels. The horizontal and vertical length in meters covered by the recording or image at a distance R is given by width and height, respectively.

**R (m)**	**Width (m)**	**Number of pixels per meter**			
		**Horizontal pixels**			
		**320**	**640**	**800**	**1024**

1	0.93	343.12	686.24	857.80	1097.99
10	9.33	34.31	68.62	85.78	109.80
50	46.63	6.86	13.72	17.16	21.96
100	93.26	3.43	6.86	8.58	10.98
200	186.52	1.72	3.43	4.29	5.49
500	466.31	0.69	1.37	1.72	2.20

**R (m)**	**height (m)**	**Vertical pixels**			
		**240**	**480**	**600**	**768**

1	1.24	193.01	386.01	482.51	617.62
10	12.43	19.30	38.60	48.25	61.76
50	62.17	3.86	7.72	9.65	12.35
100	124.35	1.93	3.86	4.83	6.18
200	248.70	0.97	1.93	2.41	3.09
500	621.74	0.39	0.77	0.97	1.24
